# Interaction of GPER-1 with the endocrine signaling axis in breast cancer

**DOI:** 10.3389/fendo.2025.1494411

**Published:** 2025-01-24

**Authors:** Luis Molina Calistro, Yennyfer Arancibia, Marcela Alejandra Olivera, Sigrid Domke, Rodrigo Flavio Torres

**Affiliations:** ^1^ Facultad de Medicina y Ciencia, Universidad San Sebastián, Puerto Montt, Chile; ^2^ Facultad de Ciencias para el cuidado de la salud, Universidad San Sebastián, Puerto Montt, Chile

**Keywords:** GPER-1, estrogen, hypothalamic-pituitary-gonadal axis, breast cancer, endocrine resistance, personalized medicine

## Abstract

G Protein-Coupled Estrogen Receptor 1 (GPER-1) is a membrane estrogen receptor that has emerged as a key player in breast cancer development and progression. In addition to its direct influence on estrogen signaling, a crucial interaction between GPER-1 and the hypothalamic-pituitary-gonadal (HPG) axis has been evidenced. The novel and complex relationship between GPER-1 and HPG implies a hormonal regulation with important homeostatic effects on general organ development and reproductive tissues, but also on the pathophysiology of cancer, especially breast cancer. Recent research points to a great versatility of GPER-1, interacting with classical estrogen receptors and with signaling pathways related to inflammation. Importantly, through its activation by environmental and synthetic estrogens, GPER-1 is associated with hormone therapy resistance in breast cancer. These findings open new perspectives in the understanding of breast tumor development and raise the possibility of future applications in the design of more personalized and effective therapeutic approaches.

## 1 Introduction

Breast cancer is a malignant disease that originates in the cells of the breast tissue. In this type of cancer, breast cells multiply abnormally and uncontrollably, forming a tumor or mass in the breast. Breast cancer comprises a variety of subtypes, and its aggressiveness and behavior can vary significantly between patients, often posing clinical challenges in terms of diagnosis and treatment (1). Morphologically, the most common type of breast cancer affects the ducts responsible for milk transport (ductal cancer), while the second most frequent form starts in the lobules, i.e. the milk-producing glands (lobular cancer).

Breast cancer is the most common form of cancer in women (2) but can also affect men (3). Conventional biomarkers used to assess the disease include estrogen (ER) and progesterone receptors (PR), as well as evaluation of the Human Epidermal Growth Factor Receptor 2 (HER2) and Epidermal Growth Factor Receptor (EGFR) genes, along with the BRCA1 and BRCA2 genes. In addition, biomarkers such as Ki-67 and p53 provide additional information on tumor aggressiveness (4). The presence or absence of the ERα receptor in the tumor cell allows the cancer’s categorization as sensitive or insensitive to estradiol, respectively. This categorization is critical for medical decisions in the process of diagnosis and treatment.

Although timely diagnosis and the development of effective therapies have led to significant progress towards reducing breast cancer mortality. The molecular variability, within and between patients (1), underlie phenotypic and behavioral changes in tumor cells. These changes drive the cellular resistance to anticancer therapies, rendering breast cancer as one of the worldwide leading causes of cancer-related deaths (5). Hence, new biomarkers that contribute to improving the diagnostic and treatment of breast cancer patients represents a major goal in breast cancer research.

**Figure 1:**
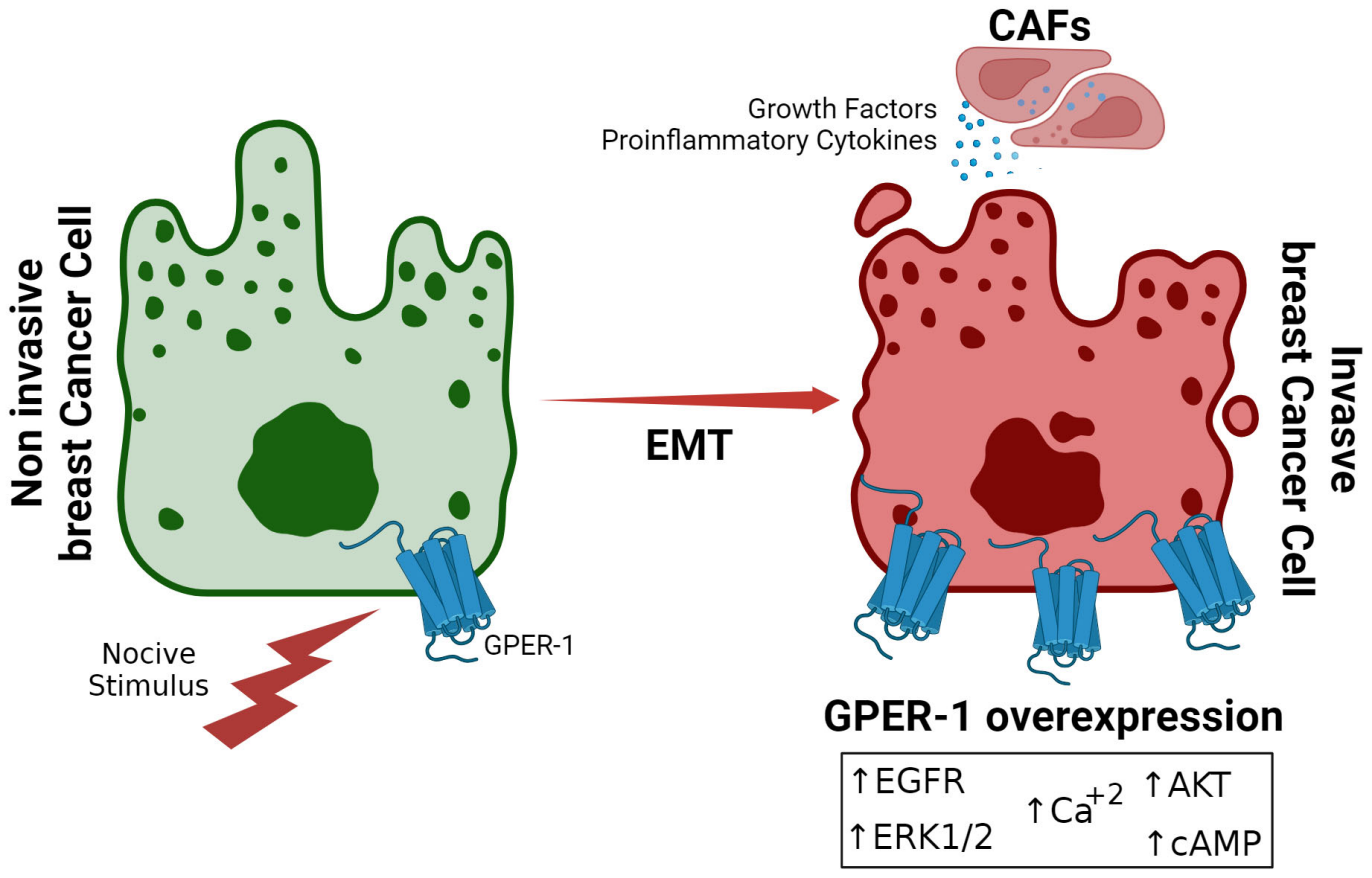
Overexpression of GPER-1 during Epithelial-Mesenchymal Transition in Breast Cancer. Prolonged exposure to noxious stimuli, such as xenoestrogens or other estrogenic molecules, creates a favorable tumor environment for epithelial-mesenchymal transition in breast cancer cells. This is characterized by increased expression of GPER-1 in the tumor cell, leading to increased activity of signaling pathways dependent on this receptor.

The Hypothalamus-Pituitary-Gonads (HPG) axis promotes organic development, contributing to reproductive function, as well as to the menstrual cycle and breast development in women. An important part of these physiological responses is mediated by estradiol through its nuclear specific receptors, ERα and ERβ. However, interaction of estradiol with the membrane G Protein Coupled Estrogen Repector-1 (GPER-1) contributes to the physiological regulation of the HPG axis, sexual hormone levels and to the fine mechanisms of estradiol release (6). In fact, the expression of GPER-1 has been detected in key tissues in human hormonal communication, such as the hypothalamus, the pituitary gland, the gonads (especially the ovaries) and the mammary gland (**Figure 1**). Importantly, alterations in the regulation of the neuroendocrine axis generate relevant effects on breast cancer development (7). In some cases, GPER-1 is overexpressed in tissues within the HPG axis, dysregulating the estrogenic signaling pathways that relay on GPER-1 receptor activity. These alterations ultimately contribute to cancer cell proliferation (8, 9). GPER-1 is also expressed in breast cancer stem cells. Cancer stem cells exhibit stem cell-like properties in terms of self-renewal and differentiation. These cells contribute to tumor growth, metastasis, and resistance to therapeutic treatments (10). A study using xenografts derived from patients with ER-/PR+ breast cancer (CSCM) has shown that GPER-1 is significantly expressed in these cells. In fact, GPER-1 silencing reduces the pluripotency characteristics of this cell type. Moreover, activation of GPER-1 by tamoxifen promotes Protein Kinase A (PKA)/Bcl-2-antagonist of cell death (BAD) phosphorylation, which maintains stemness and viability characteristics in CSCMs (Y.-T. 11).

Since the identification of GPER-1 as an estrogen receptor, it has attracted increasing interest due to its involvement in the pathophysiology of various chronic diseases, including metabolic, nervous, cardiovascular and cancer diseases (12). Interestingly, GPER-1 also interacts with molecules that exhibit structural homology with estradiol, which could contribute to breast cancer development and its relation to environmental pollution (13).

In summary, recent research indicates that the involvement of GPER-1 in the regulation of the HPG axis is extremely relevant in the context of breast cancer, as alterations in hormonal signaling promote tumor growth and cancer progression. Understanding these interactions may provide crucial information for developing personalized therapeutic strategies.

## 2 The emerging role of GPER-1 in estradiol-dependent signaling

The discovery GPER-1, also known as GPR30, in the 2000’s marked an important milestone in estrogenic signaling research (14). This finding has provided a novel insight into how estrogens interact with cells, allowing a greater understanding of estrogen versatile role in health and disease.

Early indications about GPER-1 activity involved the observation of rapid intracellular responses to estrogen (15) included calcium mobilization and activation of protein kinases. Both processes occur within seconds or minutes, contrasting to the slower genomic responses that require regulation of gene transcription driven by the activation of estrogen nuclear receptors (16).

GPER-1 is now known to play a crucial role in intracellular signal transduction in response to estradiol (17β-estradiol or E2), but also to other types of physiological estrogens, such as estrone (E1) and estriol (E3) (17). Moreover, activation of GPER-1 by several estrogenic compounds of natural (such as phytoestrogens) and synthetic origin (such as bisphenols) has also been reported (18). In addition, tools such as the GPER-1 specific synthetic agonist, G1, together with the antagonist compounds G15 and G36, have been used to assess GPER-1 function in different cells and animal models (17, 19). These compounds are derived from quinolones, and their functional groups give them bioactive properties. Since their discovery, these pharmacological tools have been essential for the development of new strategies focused on the characterization of GPER-1 signaling (10, 20, 21). New computational techniques have enabled detailed ligand analysis and facilitated the design of new drugs targeting GPER-1. This has contributed significantly to the understanding of the underlying molecular mechanisms, as well as to the identification of potential modulators and therapeutic candidates (22).

In triple-negative MDA-MB-231 and HCC 1386 cells, GPER-1 silencing using a specific siRNA reduces the invasiveness of breast tumor cells. Furthermore, this silencing increases sensitivity to tamoxifen through estrogen receptor beta (23). A recent investigation in estradiol-sensitive breast cancer cell lines resistant to 4-hydroxytamoxifen (4-OHT), the major metabolite derived from tamoxifen, showed that silencing of Cysteine-Rich Angiogenic Inducer 61 (CYR61) expression resulted in a significant decrease in cell invasion and re-sensitization to 4-OHT, suggesting that CYR61 suppression could be a promising therapeutic strategy to improve the treatment of tamoxifen-resistant breast cancer (24).

GPER-1 can mediate both genomic and non-genomic responses. Its activation leads to diverse intracellular events, such as transactivation of the epidermal growth factor receptor (EGFR) (25). EGFR transactivation leads to the rapid of mitogen-activated protein kinases (MAPKs), especially extracellular signal-regulated kinases 1 and 2 (ERK1/2), phosphorylation of phospholipase C (PLC) and phosphatidylinositol- 3-kinase (PI3K). Ultimately, adenyl cyclase (AC) stimulation directs the intracellular mobilization of Ca^2+^ (26, 27). EGFR is a key player in the regulation of cell proliferation and survival. Its interaction with GPER-1 triggers a signaling cascade involving the activation of kinases which can modulate the activity of ryanodine channels (RyR1 and RyR2) of the endoplasmic reticulum (28). Subsequent intracellular calcium release plays a crucial role in cell proliferation and in the acquisition of drug-resistant phenotypes in tumor cells, making this pathway a promising therapeutic target (29). In addition, GPER-1 regulates estradiol-related gene expression through activation of PI3K and pERK1/2, mediating cell survival and proliferation signals (30).

GPER-1 activation complexly modulates the expression of multiple microRNAs in breast cancer (31). MicroRNAs (miRNAs) are small non-coding RNA molecules that regulate gene expression at the post-transcriptional level by binding to messenger RNAs (mRNAs) and inhibiting their translation or promoting their degradation. For example, miRNAs such as miR-9-5p, miR-10b-5p and miR-21-5p are overexpressed and act as oncogenes, promoting GPER-1 expression and suppressing tumor genes such as PTEN and TIMP3, which in turn are associated with increased resistance to treatments (32–34). On the other hand, miRNAs such as miR-205-5p and miR-206 exert tumor suppressor effects by inhibiting oncogenic signaling pathways such as Ras/Raf/MEK/ERK and reducing the invasiveness of cancer cells (35). These findings could be useful not only for the development of new therapeutic strategies, but also for understanding treatment failure in cancer patients.

On the other hand, it is well established that ERα plays a gravitating role in the development of breast cancer. Of note, ERα presence and activity are closely related to the growth and proliferation of tumor cells (36). Thereof, tumors expressing this receptor are typed as ERα-positive, meaning that they are stimulated by estradiol, the most potent biological form of estrogen (4). These cancers correspond to 60%-70% of breast cancer cases (37). On the other hand, overexpression of ERα favors the stabilization and repair of the genome of tumor cells (38, 39). The ERα-positive classification is relevant for choosing a therapeutic strategy. Tamoxifen or aromatase inhibitors are the general choice in these cases, as the objective is to block or reduce ERα activity. ERα-negative breast tumors do not respond to hormone therapy and tend to grow and proliferate more rapidly, have a higher propensity to metastasize, and have a limited response to chemotherapy, resulting in a less favorable prognosis.

## 3 GPER-1 in the communication of the hypothalamic-pituitary-gonadal axis, new implications in breast cancer.

### 3.1 Role of GPER-1 in the endocrine regulatory axis HPG

The ubiquity of GPER-1 in various body tissues suggests its fundamental role in organ homeostasis and dysregulation. Numerous studies have demonstrated its involvement in the regulation of key physiological systems, such as the cardiovascular and immune systems. For a comprehensive review of the multiple functions of GPER-1 in these contexts, please refer to (12). Until recently, the cellular response to estradiol in the nervous and reproductive systems were thought to rely exclusively on the classical nuclear receptors. However, the discovery of GPER-1 has revealed that a significant part of estrogenic responses may result from the activity of this membrane receptor (12). Importantly, the responses commanded by GPER-1 may be different in males and females, due to changes in their expression levels, especially during the estrous cycle (40).

GPER-1 has been identified at various locations in the central nervous system, suggesting a broad involvement of this receptor in both behavioral and reproductive processes. For instance, GPER-1 has been detected in different hypothalamic cell types, including neurons, astrocytes, and oligodendrocytes (41).

Furthermore, studies in female rats have determined that GPER-1 is related to estradiol activity on the functions of the anterior hypothalamus, ranging from feeding behavior to sexual receptivity (41). Hence, this receptor could collaborate in several of the biological responses regulated by the hypothalamus, such as sleep, feeding, stress response and endocrine regulation (42). In addition, GPER-1 expression has also been detected in the amygdala and dorsal hippocampus, modulating anxiety, social recognition, and spatial memory (40). Opening the interrogation of its role in other neuronal processes.

The presence of GPER-1 has recently been determined in lactotrophs, a cell type of adenohypophysis whose main function is to synthesize the hormone prolactin. Interestingly, the GPER-1 agonist G1 induced a rapid stimulation of prolactin secretion, both *in vitro* and *ex vivo.* This effect was prevented by the GPER-1 antagonist G36 (43). Furthermore, GPER-1 is expressed in anterior pituitary gonadotroph cells. Modulating the response of these cells to gonadotropin-releasing hormone (GnRH) and contributing to the negative feedback exerted by estradiol on luteinizing hormone (LH) secretion (44).

LH plays a crucial role in the reproductive system of both males and females. In males, GnRH stimulates Leydig cells in the testis for the synthesis of testosterone, which together with follicle-stimulating hormone (FSH) promotes the process of spermatogenesis (45). GPER-1 has also been identified in testicular tissue. Somatic, Leydig and Sertoli cells, as well as germ cells, including spermatogonia, spermatocytes and spermatids show GPER-1 expression (26) (**Table 1**). Furthermore, in Leydig cells, estradiol directs a GPER-1-dependent down-regulation of testosterone synthesis (20-30%) relative to untreated Leydig cells (46). Immature Sertoli cells survival is enhanced by stimulation with estradiol or G1. Increasing anti-apoptotic signals through the GPER-1/EGFR/mitogen-activated protein kinase3/1 (MAPK3/1) pathway (47, 48). In this line, it has been observed that nanomolar concentrations of the synthetic estrogenic compound bisphenol A (BPA) increases the proliferation rate of mouse immature Sertoli cells. The increase in the proliferation of this cells involves both GPER/EGFR/ERK1/2 and ERα/β/ERK1/2 pathways (49). Altogether, these findings position GPER-1 as a mediator of the estrogen-dependent testicular development and spermatogenesis.

**Table 1:** GPER-1 and its role in the HPG axis.

System	Region/Cell	GPER-1 Function	Detailed Signaling Pathways	References
Central Nervous System	Hypothalamus	Regulation of feeding, sexual behavior, sleep, stress, hormone secretion	MAPK/ERK1/2, PI3K/Akt, Ca2+, cAMP	41, 42;
	Amygdala, Dorsal Hippocampus	Modulation of anxiety, social recognition, spatial memory	MAPK/ERK1/2, Ca2+	40
Pituitary Gland	Lactotrophs	Stimulation of prolactin secretion	MAPK/ERK1/2	43
	Gonadotrophs	Modulation of GnRH response, negative feedback on LH secretion	MAPK/ERK1/2	44
Testis	Leydig cells	Regulation of testosterone synthesis (downregulation)	MAPK/ERK1/2, EGFR	26, 46
	Sertoli cells	Survival, proliferation	MAPK/ERK1/2, EGFR, PI3K/Akt Synergistic effects with ERα/β in BPA-induced proliferation	47–49
	Germ cells	Spermatogenesis	MAPK/ERK1/2	26
Ovaries	Oocytes	Oocyte maturation	MAPK/ERK1/2	50, 57
	Granulosa cells	Collaboration with FSHR, follicular maturation, gametogenesis	MAPK/ERK1/2, PI3K/Akt Formation of heteromeric complexes with FSHR	51–53

In females, luteinizing hormone (LH) stimulates ovulation and subsequent corpus luteum formation (54). The involvement of GPER-1 in ovogenesis has been the subject of study in several vertebrate species (55, 56). For instance, GPER-1 expression has been detected in the oocyte membrane, especially as it reaches a higher degree of maturation (50). More recently, follicle stimulating hormone (FSH) has been found to stimulate aromatase enzyme expression and estradiol biosynthesis in mouse cumulus-oocyte complexes (COCs). Estradiol then activates the GPER-1/ERK1/2 pathway promoting oocyte expansion and maturation (57).

Granulosa cells are crucial components of ovarian follicles, surrounding and providing nutritional support to developing oocytes. Interestingly, expression of both FSH receptor (FSHR) along with GPER-1 has been demonstrated in this cell type (51). During follicular maturation, in response to FSH released by the adenohypophysis, granulosa cells convert androgens to estradiol for regulation of the menstrual cycle and preparation of the uterus for potential implantation. Recently, the formation of heteromeric complexes between GPER-1 and FSHR at the cell membrane has been demonstrated, contributing to the viability of granulosa cells (52). GPER-1 and FSHR are estimated to collaborate by generating a signaling network that promotes gametogenesis (53).

Although mice lacking GPER-1 do not show clear alterations in reproduction or fertility (58), the evidence indicates that GPER-1 contributes to the synchronization of sex hormone release, particularly estradiol, modulating its physiological effects on peripheral and reproductive tissues. On the other hand, the GPER- 1 deficient murine model allowed linking this receptor to the development and metastatic capacity of breast cancer (59).

Several investigations have evidenced the impact of various compounds with estrogenic activity on the hypothalamic-pituitary axis, although the interaction of these compounds with GPER-1 is not entirely clear, some studies suggest an active role of GPER-1 in the hypothalamic-pituitary axis, in the context of exposure to molecules with estrogenic activity (60), opening the possibility of new avenues of research on unconventional mechanisms of hormone action (61). In addition, bisphenol-GPER-1 interaction has been associated with male infertility (62). The results of future studies could reveal complex molecular mechanisms and their implications in endocrine pathophysiology.

### 3.2 GPER-1 in the endocrine disruption and pathophysiology of breast cancer

GPER-1 also is expressed in different types of mammary cells, including epithelial cells, myoepithelial cells, and stromal cells, being involved in normal mammary gland development and function (63). In addition, GPER-1 has also been observed to be associated with several pathological processes, especially breast cancer.

Aging is considered one of the main risk factors for breast cancer development (64). With age, the ability to repair DNA decreases, making cells prone to cancer-promoting genetic changes. In addition, the ability of the immune system to respond to tumor cells is altered during aging (65). Another important factor corresponds to alterations in the regulation of hormone release. During menopause, which marks the end of menstruation, the ovaries decrease the biosynthesis of sex hormones (66). However, during a time corresponding to the menopausal transition (MT), the adenohypophysis generates a monotropic (constant) increase in FSH in response to the ovarian reserve reduction. During MT estradiol levels also increase, before decreasing significantly during menopause (67). However, not all estrogenic hormones are downregulated during this period. One example is estrone, which is mainly produced in adipose tissue. Hence, the decrease in estradiol during menopause does not mean a total reduction in estrogen exposure (**Figure 1**).

Another important factor in breast cancer development is the regulation exerted by the tumor microenvironment. Tumor microenvironment may promote carcinoma cells to change their epithelial nature to mesenchymal characteristics, a phenomenon known as epithelial-mesenchymal transition (EMT) (68). Importantly, recent research indicates that estrone induces EMT, thus facilitating the invasiveness of breast cancer (69). Furthermore, it has been suggested that, in postmenopausal women, the relationship between estrone and estradiol may be an important factor in breast cancer risk (69). Hence, estrone, by acting as a GPER-1 agonist (70), could contribute to the development of estrogen-sensitive breast cancer. GPER-1 activation correlates with increased expression of mesenchymal markers such as vimentin and N-cadherin (71). In turn, estrone, a major GPER-1 agonist (17), has been implicated in promoting EMT (69). Exposure of breast cancer cells to estrone induces the expression of EMT-associated transcription factors, such as Snail and Slug, and promotes the generation of more invasive cells (Y. 72). These findings suggest a causal relationship between GPER-1 activation by estrone and EMT induction, underscoring the potential role of this receptor in tumor progression and metastasis. Furthermore, pharmacological inhibition of GPER-1 or inhibition of its expression by interfering RNA (siRNA) techniques reverses EMT and reduces the invasiveness of cancer cells (73).

On the other hand, hormone replacement therapy, aimed at preserving the beneficial effects of estradiol on female physiology. Particularly regarding metabolism and cardiovascular health (74), could also have undesirable side effects, contributing to the development of breast cancer (75). Similarly, the use of oral contraceptives, consisting of a combination of estrogens and progestogens, is considered a risk factor for the development of breast cancer (76). Increased exposure to estrogens alters the physiological regulation of sex hormone release, disrupting the signaling commanded by estrogen receptors and promotes hormone-dependent cancer development (77). In fact, it has been observed that a significant number of estradiol-sensitive (ERα-positive) breast cancer cases co-express GPER-1 (78), which is associated with worse prognosis and diminished survival of patients, even in those patients treated with tamoxifen (78, 79).

Dysregulation of estrogen signaling may play a critical role in tumor progression by providing an environment conducive to tumor growth and facilitating metastasis (80). Increased expression of GPER-1, as well as its aberrant activation, is associated with several hormone-dependent cancers, including cervical (81), prostate (82), testicular (26), breast (83), lung (81) and glioblastoma (84). However, in some types of reproductive tumors, antitumor activity of the GPER-1 receptor has been demonstrated through mechanisms such as apoptosis, cell cycle and arrest in G2 (85). Low levels of GPER-1 are associated with antitumor effects in prostate cancer (86). Interestingly, an overexpression of GPER-1 in ovarian cancer is associated with decreased tumor development (87).

Some research indicates that GPER-1 expression is strongly influenced by epigenetic factors, especially through DNA methylation. This process implies that proteins that bind methyl groups can recruit both activators and repressors, particularly on CpG islands, which are regions rich in cytosine and guanine dinucleotides. These islands are often found at transcription start sites (88). Two CpG islands are associated with GPER. One, located at approximately 1 kb upstream of the transcription initiation site, has been associated with the transcriptional regulation of GPER-1. Interestingly, breast cancer cell lines that express GPER-1 showed hypomethylation of this CpG island. Furthermore, treatment with 5-azacytidine, an inhibitor of DNA methyltransferases, increased GPER-1 expression (89). Suggesting an inverse relation between DNA methylation and GPER-1 expression in breast cancer (90). Similar results have been observed for gastric cancer (91) and colorectal cancer, with samples from patients showing higher methylation levels and lower GPER1 expression compared to patient-matched normal tissue (92). Recently, analysis of various databases has shown that DNA methylation of GPER-1 and ERα is associated with survival in tumor patients. It is suggested that methylation of these genes may play a role in cancer progression by modulating chromatin configuration (93).

Additionally, molecules that can mimic the biological effects of estrogens due to a structural homology with estradiol are globally cataloged as xenoestrogens. Environmental pollution determines our constant exposition to these molecules with estrogenic capacity. Raising interest in the association between xenoestrogens exposition and cancer development (94). Xenoestrogens are foreign to our physiology, some of these molecules have their origin in plants, as is the case of phytoestrogens, and others are of industrial origin, covering many molecules from phthalates to bisphenols (18). Much of the evidence indicates that industrial xenoestrogens may act as endocrine disruptors (18, 94, 95). In this context, bisphenol A (BPA) and phthalates, a chemical compound that is incorporated in plastic containers used to store water, beverages, food, and numerous items of modern life, stands out (96). Phthalates, BPA, and other types of bisphenols have been detected in virtually all biological fluids and tissues. These include amniotic fluid (97) and adipose tissue (98–100).

Phthalates, such as butylbenzyl phthalate (BBP), dibutyl phthalate (DBP) and di(2-ethylhexyl)phthalate (DEHP), have been shown to have estrogenic effects in breast cancer cells, interacting with estrogen receptor alpha (ERα) at micromolar concentrations (101, 102). Although their ability to induce cell proliferation suggests a possible interaction with GPER-1, direct binding between these phthalates and GPER-1 has not yet been demonstrated (102).

Environmentally relevant doses of BPA generate activation of classical estrogen receptors, inducing protumor activity (103). It has recently been proposed that stimulation of estradiol-sensitive breast cancer cells with BPA increases breast cancer cell proliferation (104). However, it has also been determined that BPA can exacerbate cancer cell behavior by acting on G protein-coupled receptors, specifically GPER-1 (13, 105) (**Table 2**).

**Table 2:** Factors contributing to breast cancer development.

Factor	Description	Impact on Breast Cancer Development	Signaling Pathways	References
Age-related Hormonal Changes	Aging, menopause, hormonal fluctuations (Estrogens, FSH)	Altered hormonal balance, increased breast cancer risk, especially hormone-dependent types	Estrogen receptors, GPER-1 → MAPK ERK1/2, EGFR, PI3K/Akt, Ca2+ release	17, 64–67, 69–71
Endocrine Disruption by Exogenous Compounds	Hormone therapy, contraceptives, xenoestrogens (BPA, BPS, BPAF, phthalates). Epigenetic regulation of GPER-1 (DNA methylation)	Altered hormonal balance, increased breast cancer risk, especially hormone-dependent types; Epigenetic modifications influence GPER-1 expression. Tamoxifen-mediated GPER-1 activation sustains CSCM stemness and viability.	Estrogen receptors, GPER-1 → MAPK ERK1/2, EGFR, PI3K/Akt, Ca2+ release. Upregulating PKA/BAD phosphorylation.	11, 13, 18, 75, 76, 83, 88–95, 103, 105–109
Tumor Microenvironment and GPER-1 Activation	Cell-cell interactions, extracellular matrix; GPER-1 activation correlates with mesenchymal markers (vimentin, N-cadherin). Estrone induces EMT via Snail and Slug transcription factors	Promotes cell proliferation, EMT, invasion, angiogenesis, and therapy resistance; creates a pro-tumor microenvironment	GPER-1 → MAPK ERK1/2, EGFR, PI3K/Akt, Ca2+ release; activation of CAFs → IL-6, VEGF, other growth factors	9, 17, 69, 69, 71–73, 85, 110, 111

The pathophysiological effects of different concentrations of BPA have been analyzed. High doses, in the micromole range, have been linked to oxidative stress, subcellular damage, cytotoxicity, and apoptosis (112). Chronic exposure to these high doses may facilitate inflammation, pancreatic beta-cell death and metabolic dysfunction (113, 114). However, low doses, in the nanomoles range, have raised more concern (115) due to their prevalence in the environment (116) and the variability of BPA in serum, ranging from 1 to 10 nM, which has a high potential to alter endocrine function (117). This alteration can interfere with the gonadotropin-releasing hormone (GnRH) release axis, generating alterations in early development and in the human reproductive cycle (118).

In murine models, low oral doses of BPA have shown remarkable proestrogenic activity (119). Several studies indicate that low concentrations of BPA can modify cell behavior in prostate (120) and mammary (121) tissues, which could increase long-term cancer risk. Additionally, exposure to BPA and other endocrine disruptors has adverse effects on genes that regulate placental function and fetal development (122, 123), associated with negative consequences on fetal development and neurological function (122).

Due to negative health effects and growing concern in the scientific community and the general public, the use of other bisphenols, such as bisphenol S (BPS) and bisphenol AF (BPAF), has been promoted (99). However, these compounds exhibit hormonal properties, with BPAF being more potent than BPS. In fact, several reports, using as models yeast (*Saccharomyces cerevisiae*), zebrafish (*Danio rerio*), or human and rat stem cells, indicate that their toxic and estrogenic effects are similar or even exceed those of BPA (106–108).

In MCF-7 cells, low concentrations of BPAF through GPER-1 triggered PI3K/Akt and ERK1/2 signaling pathways, promoting cell proliferation, and increased levels of intracellular calcium, and reactive oxygen species (ROS) (109). Recently, it has been observed in immortalized murine hypothalamic cells, both of embryonic and adult origin, that exposure to BPS, through GPER-1, induces the expression of the Agouti-related peptide (AgRP) gene, a neuropeptide crucial in the regulation of appetite and energy balance, which could contribute to metabolic disorders associated with obesity (124).

Adipose tissue tends to bioaccumulate various types of xenoestrogens, due to the lipophilic characteristics of these compounds (125, 126). This phenomenon, in the case of phthalates and bisphenols, has been consistently linked to adipogenesis, and to the long-term development of endocrine and metabolic diseases (127–129).

**Figure 2:**
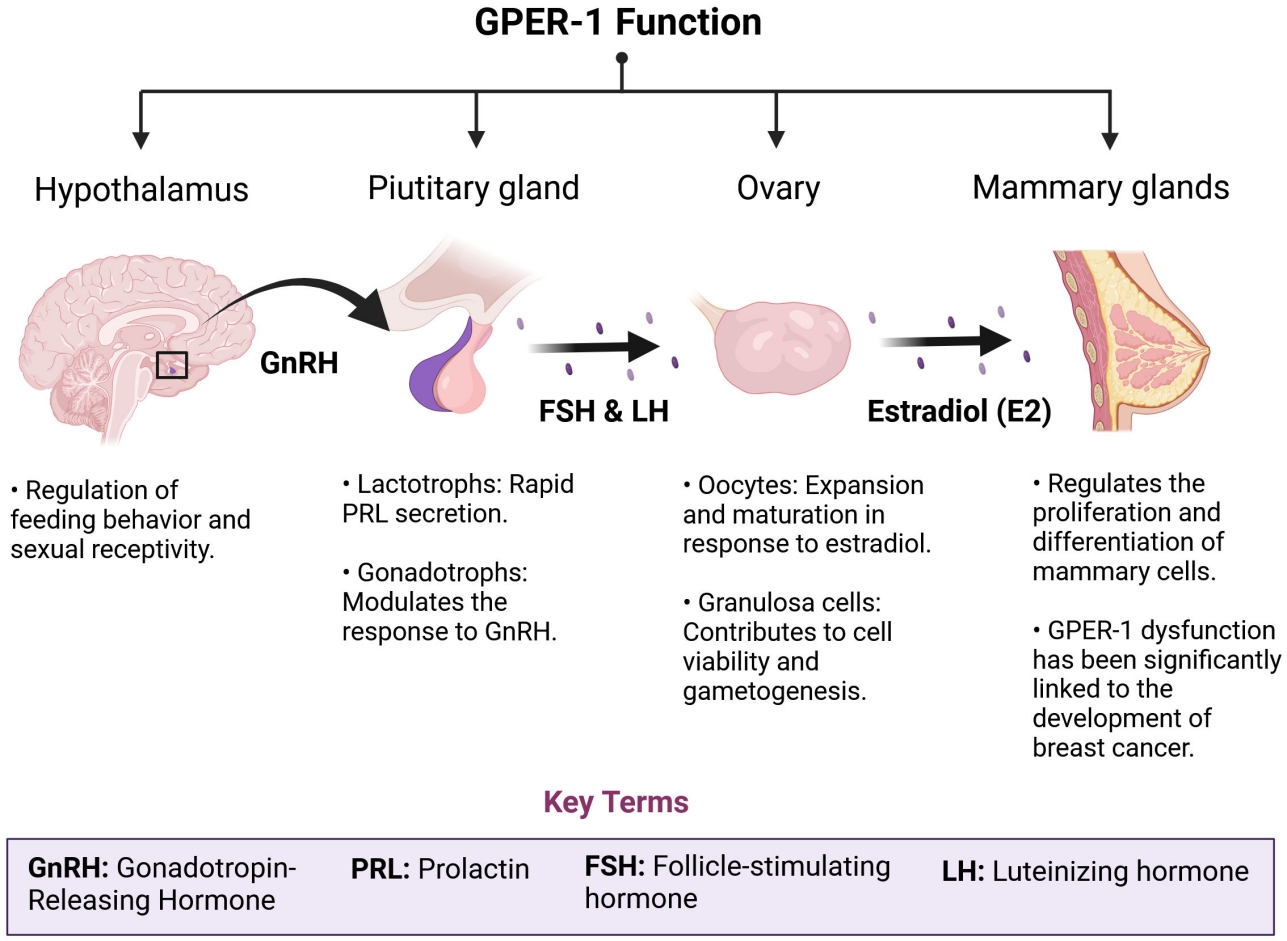
GPER-1 plays a crucial role in the physiological regulation of the HPG axis. GPER-1 is involved in the intricate regulatory network of the hypothalamic-pituitary-gonadal (HPG) axis. The ubiquity of this receptor, both in the central nervous system and in peripheral tissues, determines an integrative role of neuroendocrine and environmental signals. In the hypothalamus and pituitary, GPER-1 modulates the synthesis and pulsatile release of gonadotropins, hormones essential for follicular development, ovulation and spermatogenesis. In the ovaries, GPER-1 mediates the effects of estradiol on cell proliferation, apoptosis and steroid synthesis, thus influencing ovarian function and fertility. The mammary gland, another target tissue of GPER-1, undergoes morphological and functional changes in response to hormonal fluctuations. Disruption of GPER-1 signaling by exposure to estrogenic chemicals or physiological alterations such as menopause can trigger a cascade of events leading to reproductive, metabolic and carcinogenic disorders.

In perspective, exposure to hormone replacement therapy, contraceptive, or xenoestrogens triggers intracellular signaling pathways that are mediated by GPER-1 and induced by physiological estrogens (30). However, in the context of breast cancer, these pathways may exacerbate tumoral behavior, enhancing the signaling pathway activation or its components, such as ERK1/2, AKT, cyclic adenosine monophosphate (cAMP) or by increasing intracellular calcium levels. GPER-1 may also act through direct or indirect association with other estradiol-responsive receptors or inflammation-related receptors, and its levels may also be affected by the activity of cancer-associated fibroblasts (CAFs) in the tumor microenvironment (**Figure 2**). Thus, for example, continuous exposition to tamoxifen, the first-line drug against estradiol-sensitive breast cancer, overexpresses GPER-1, increasing calcium mobilization and cell proliferation (9). Suggesting that GPER-1 overexpression constitutes a mechanism of drug resistance (9, 110, 111).

Interestingly, a recognition domain of GPER-1 in ERα has recently been reported, such a region has also been found in a truncated isoform of estrogen receptor alpha, named as ERα36 (130). An association between GPER-1 and insulin-like growth factor (IGF1R) signaling, promoting breast cancer metastasis, has also been suggested (131). Similarly, a relationship of GPER-1 with proinflammatory receptors has been observed (9, 132). Further research is required to increase our understanding of the interactions between GPER-1 and other receptors, and the role those interactions play in breast cancer development.

GPER-1 expression has also been observed in triple-negative breast cancer (TNBC) (83), a neoplasm characterized by a lack of ERα, PR and HER2 receptors. In this context, GPER-1 can modulate key pathways, including MAPK activation and EGFR signaling (133), contributing to cell proliferation and invasion. This scenario associates TNBC with the most aggressive phenotypes of breast cancer (83). Indeed, the RAS/RAF/MEK/ERK signaling cascade, key in the physiological response to estradiol, has been found to be frequently over activated in various types of cancers, although mutations in this pathway are not usually described in breast cancer. TNBC cancer has been associated to driver mutations in the Kirsten Rat Sarcoma Viral Oncogene Homolog (KRAS), and v-Raf murine sarcoma viral oncogene homolog B (BRAF) genes, promoting the synthesis of K-RAS and RAF proteins (134). Furthermore, a recent report used CRISPR/Cas9 to knockout GPER-1 expression in triple-negative MDA- MB-231 cells. Cells lacking GPER-1 showed a shift towards pro-apoptotic and antiproliferative signaling driven by reduced cAMP levels and activation of the *c*-*Jun* N-terminal kinase (JNK/c- Jun)/p53/Noxa pathway (135). Therefore, GPER-1 would be intrinsically related to the mechanisms that determine the development of TNBC.

Additionally, in the tumor microenvironment, GPER-1 expression has been detected in CAFs. GPER-1 activation stimulates the secretion of proinflammatory factors such as interleukin 6 (IL-6) and epidermal growth factor (VEGF) (**Figure 2**). This phenomenon would ultimately also contribute to resistance to hormonal treatments such as tamoxifen (85).

Taken together, these data suggest that GPER-1 modulates a complex signaling network of importance for the development of estradiol-sensitive breast cancer and TNBC, which is influenced by several interrelated factors. First, GPER-1 expression and tumor cell type are critical, as GPER-1 shows remarkable versatility in the target signaling pathways it activates, which generates a variable impact depending on the cellular context. Second, the tumor microenvironment plays a crucial role; the extracellular matrix can modify GPER-1 activity and thus alter tumor responses. Third, activation of GPER-1 by molecules that mimic estrogen structure, such as xenoestrogens (e.g., Bisphenol A), poses a potential risk of endocrine disruption. Finally, the interaction of GPER-1 with other receptors, such as EGFR and ERα, may amplify estrogenic signaling, opening exciting opportunities to investigate combination therapies targeting these pathways.

## 4 Conclusion

The dynamic interaction between GPER-1 and signals from the hypothalamus-pituitary- gonads axis suggests a direct connection between sex hormone regulation and molecular events associated with the progression of several types of cancer, especially breast cancer. The ability of GPER-1 to modulate key signaling pathways, influence gene expression, and participate in specific molecular cascades in nervous and reproductive tissue is a developing area but represents a significant advance toward a greater understanding of the pathophysiology of breast cancer and other chronic nerve and metabolic diseases.

Recent discoveries about the interaction of GPER-1 with synthetic and environmental estrogens emphasize the importance of considering the expression and activity of this receptor in the formulation of more effective and specific therapeutic approaches for breast cancer, establishing an additional link that strengthens the ability to tailor therapeutic interventions to the specific molecular characteristics of each patient.

## References

[1] Yersal O Barutca S. Biological subtypes of breast cancer: Prognostic and therapeutic implications. World J Clin Oncol. (2014) 5:412–24. doi: 10.5306/wjco.v5.i3.412 PMC4127612 25114856

[2] Łukasiewicz S Czeczelewski M Forma A Baj J Sitarz R Stanisławek A. Breast cancer-epidemiology, risk factors, classification, prognostic markers, and current treatment strategies-an updated review. Cancers. (2021) 13:4287. doi: 10.3390/cancers13174287 34503097 PMC8428369

[3] Fox S Speirs V Shaaban AM. Male breast cancer: An update. Virchows Archiv: Int J Pathol. (2022) 480:85–93. doi: 10.1007/s00428-021-03190-7 34458944

[4] Mueller C Haymond A Davis JB Williams A Espina V. Protein biomarkers for subtyping breast cancer and implications for future research. Expert Rev Proteomics. (2018) 15:131–52. doi: 10.1080/14789450.2018.1421071 PMC6104835 29271260

[5] Arnold M Morgan E Rumgay H Mafra A Singh D Laversanne M. Current and future burden of breast cancer: Global statistics for 2020 and 2040. Breast (Edinburgh Scotland). (2022) 66:15–23. doi: 10.1016/j.breast.2022.08.010 36084384 PMC9465273

[6] Chimento A Sirianni R Casaburi I Pezzi V. Role of estrogen receptors and g protein-coupled estrogen receptor in regulation of hypothalamus-pituitary-testis axis and spermatogenesis. Front Endocrinol. (2014) 5:1. doi: 10.3389/fendo.2014.00001 PMC3893621 24474947

[7] Jiang S-H Zhang X-X Hu L-P Wang X Li Q Zhang X-L. Systemic regulation of cancer development by neuro-endocrine-immune signaling network at multiple levels. Front Cell Dev Biol. (2020) 8:586757. doi: 10.3389/fcell.2020.586757 33117814 PMC7561376

[8] Chevalier N Hinault C Clavel S Paul-Bellon R Fenichel P. GPER and testicular germ cell cancer. Front Endocrinol. (2020) 11:600404. doi: 10.3389/fendo.2020.600404 PMC7870790 33574796

[9] Molina L Bustamante F Ortloff A Ramos I Ehrenfeld P Figueroa CD. Continuous exposure of breast cancer cells to tamoxifen upregulates GPER-1 and increases cell proliferation. Front Endocrinol. (2020) 11:563165. doi: 10.3389/fendo.2020.563165 PMC7561417 33117280

[10] Visvader JE Lindeman GJ. Cancer stem cells in solid tumours: Accumulating evidence and unresolved questions. Nat Rev Cancer. (2008) 8:755–68. doi: 10.1038/nrc2499 18784658

[11] Chan Y-T Lai AC-Y Lin R-J Wang Y-H Wang Y-T Chang W-W. GPER-induced signaling is essential for the survival of breast cancer stem cells. Int J Cancer. (2020) 146:1674–85. doi: 10.1002/ijc.32588 PMC7003894 31340060

[12] Prossnitz ER Barton M. The G protein-coupled oestrogen receptor GPER in health and disease: An update. Nature Reviews. Endocrinology. (2023) 19:407–24. doi: 10.1038/s41574-023-00822-7 PMC10187525 37193881

[13] Molina L Figueroa CD Ehrenfeld P. Interaction of bisphenol A with G protein: coupled receptors - new paradigms in breast cancer. En Bisphenols. (2021) 1–25. doi: 10.5772/intechopen.101204

[14] Filardo EJ Quinn JA Bland KI Frackelton AR. Estrogen-induced activation of Erk-1 and Erk-2 requires the G protein-coupled receptor homolog, GPR30, and occurs *via* trans-activation of the epidermal growth factor receptor through release of HB-EGF. Mol Endocrinol (Baltimore Md.). (2000) 14:1649–60. doi: 10.1210/mend.14.10.0532 11043579

[15] Szego CM Davis JS. Adenosine 3’,5’-monophosphate in rat uterus: Acute elevation by estrogen. Proc Natl Acad Sci United States America. (1967) 58:1711–8. doi: 10.1073/pnas.58.4.1711 PMC223984 4295833

[16] Revankar CM Cimino DF Sklar LA Arterburn JB Prossnitz ER. A transmembrane intracellular estrogen receptor mediates rapid cell signaling. Sci (New York N.Y.). (2005) 307:1625–30. doi: 10.1126/science.1106943 15705806

[17] Prossnitz ER Arterburn JB. International union of basic and clinical pharmacology. XCVII. G protein-coupled estrogen receptor and its pharmacologic modulators. Pharmacol Rev. (2015) 67:505–40. doi: 10.1124/pr.114.009712 PMC4485017 26023144

[18] Wang X Ha D Yoshitake R Chan YS Sadava D Chen S. Exploring the biological activity and mechanism of xenoestrogens and phytoestrogens in cancers: emerging methods and concepts. Int J Mol Sci. (2021) 22:8798. doi: 10.3390/ijms22168798 34445499 PMC8395949

[19] Duarte-Guterman P Lieblich SE Chow C Galea LAM. Estradiol and GPER activation differentially affect cell proliferation but not GPER expression in the hippocampus of adult female rats. PLoS One. (2015) 10:e0129880. doi: 10.1371/journal.pone.0129880 26075609 PMC4468121

[20] Dennis MK Burai R Ramesh C Petrie WK Alcon SN Nayak TK. *In vivo* effects of a GPR30 antagonist. Nat Chem Biol. (2009) 5:421–7. doi: 10.1038/nchembio.168 PMC2864230 19430488

[21] Dennis MK Field AS Burai R Ramesh C Petrie WK Bologa CG. Identification of a GPER/GPR30 antagonist with improved estrogen receptor counterselectivity. J Steroid Biochem Mol Biol. (2011) 127:358–66. doi: 10.1016/j.jsbmb.2011.07.002 PMC3220788 21782022

[22] Grande F Occhiuzzi MA Lappano R Cirillo F Guzzi R Garofalo A. Computational approaches for the discovery of GPER targeting compounds. Front Endocrinol. (2020) 11:517. doi: 10.3389/fendo.2020.00517 PMC7417359 32849301

[23] Schmitz V Bauerschmitz G Gallwas J Gründker C. Suppression of G protein-coupled estrogen receptor 1 (GPER1) enhances the anti-invasive efficacy of selective ERβ Agonists. Anticancer Res. (2022) 42:5187–94. doi: 10.21873/anticanres.16025 36288854

[24] Bauerschmitz G Hüchel S Gallwas J Gründker C. Inhibition of increased invasiveness of breast cancer cells with acquired tamoxifen resistance by suppression of CYR61. Cancer Genomics Proteomics. (2023) 20:531–8. doi: 10.21873/cgp.20403 PMC10614060 37889058

[25] Filardo EJ. Epidermal growth factor receptor (EGFR) transactivation by estrogen *via* the G-protein-coupled receptor, GPR30: A novel signaling pathway with potential significance for breast cancer. J Steroid Biochem Mol Biol. (2002) 80:231–8. doi: 10.1016/s0960-0760(01)00190-x 11897506

[26] Chimento A De Luca A Nocito MC Avena P La Padula D Zavaglia L. Role of GPER-mediated signaling in testicular functions and tumorigenesis. Cells. (2020) 9:2115. doi: 10.3390/cells9092115 32957524 PMC7563107

[27] Filardo EJ Quinn JA Frackelton AR Bland KI. Estrogen action *via* the G protein-coupled receptor, GPR30: Stimulation of adenylyl cyclase and cAMP-mediated attenuation of the epidermal growth factor receptor-to-MAPK signaling axis. Mol Endocrinol (Baltimore Md.). (2002) 16:70–84. doi: 10.1210/mend.16.1.0758 11773440

[28] Danila CI Hamilton SL. Phosphorylation of ryanodine receptors. Biol Res. (2004) 37:521–5. doi: 10.4067/s0716-97602004000400005 15709678

[29] Panda S Chatterjee O Roy L Chatterjee S. Targeting Ca2+ signaling: A new arsenal against cancer. Drug Discovery Today. (2022) 27:923–34. doi: 10.1016/j.drudis.2021.11.012 34793973

[30] Bustamante-Barrientos FA Méndez-Ruette M Ortloff A Luz-Crawford P Rivera FJ Figueroa CD. The impact of estrogen and estrogen-like molecules in neurogenesis and neurodegeneration: beneficial or harmful? Front Cell Neurosci. (2021) 15:636176. doi: 10.3389/fncel.2021.636176 33762910 PMC7984366

[31] Petri BJ Klinge CM. Regulation of breast cancer metastasis signaling by miRNAs. Cancer Metastasis Rev. (2020) 39:837–86. doi: 10.1007/s10555-020-09905-7 PMC7487050 32577859

[32] De Mattos-Arruda L Bottai G Nuciforo PG Di Tommaso L Giovannetti E Peg V. MicroRNA-21 links epithelial-to-mesenchymal transition and inflammatory signals to confer resistance to neoadjuvant trastuzumab and chemotherapy in HER2-positive breast cancer patients. Oncotarget. (2015) 6:37269–80. doi: 10.18632/oncotarget.5495 PMC4741929 26452030

[33] Iyevleva AG Kuligina ES Mitiushkina NV Togo AV Miki Y Imyanitov EN. High level of miR-21, miR-10b, and miR-31 expression in bilateral vs. Unilateral breast carcinomas. Breast Cancer Res Treat. (2012) 131:1049–59. doi: 10.1007/s10549-011-1845-z 22057972

[34] Zhang J Yang J Zhang X Xu J Sun Y Zhang P. MicroRNA-10b expression in breast cancer and its clinical association. PLoS One. (2018) 13:e0192509. doi: 10.1371/journal.pone.0192509 29408861 PMC5800653

[35] Hasegawa T Adachi R Iwakata H Takeno T Sato K Sakamaki T. ErbB2 signaling epigenetically suppresses microRNA-205 transcription *via* Ras/Raf/MEK/ERK pathway in breast cancer. FEBS Open Bio. (2017) 7:1154–65. doi: 10.1002/2211-5463.12256 PMC5537069 28781955

[36] Clusan L Ferrière F Flouriot G Pakdel F. A basic review on estrogen receptor signaling pathways in breast cancer. Int J Mol Sci. (2023) 24:6834. doi: 10.3390/ijms24076834 37047814 PMC10095386

[37] Gopinath P Oviya RP Gopisetty G. Oestrogen receptor-independent actions of oestrogen in cancer. Mol Biol Rep. (2023) 50:9497–509. doi: 10.1007/s11033-023-08793-8 37731028

[38] Caldon CE. Estrogen signaling and the DNA damage response in hormone dependent breast cancers. Front Oncol. (2014) 4:106. doi: 10.3389/fonc.2014.00106 24860786 PMC4030134

[39] Suba Z. Estrogen regulated genes compel apoptosis in breast cancer cells, whilst stimulate antitumor activity in peritumoral immune cells in a janus-faced manner. Curr Oncol (Toronto Ont.). (2024) 31:4885–907. doi: 10.3390/curroncol31090362 PMC11431267 39329990

[40] Llorente R Marraudino M Carrillo B Bonaldo B Simon-Areces J Abellanas-Pérez P. G protein-coupled estrogen receptor immunoreactivity fluctuates during the estrous cycle and show sex differences in the amygdala and dorsal hippocampus. Front Endocrinol. (2020) 11:537. doi: 10.3389/fendo.2020.00537 PMC7426398 32849310

[41] Marraudino M Carrillo B Bonaldo B Llorente R Campioli E Garate I. G protein-coupled estrogen receptor immunoreactivity in the rat hypothalamus is widely distributed in neurons, astrocytes, and oligodendrocytes, fluctuates during the estrous cycle, and is sexually dimorphic. Neuroendocrinology. (2021) 111:660–77. doi: 10.1159/000509583 32570260

[42] Hajdarovic KH Yu D Webb AE. Understanding the aging hypothalamus, one cell at a time. Trends Neurosci. (2022) 45:942–54. doi: 10.1016/j.tins.2022.10.004 PMC9671837 36272823

[43] Camilletti MA Abeledo-MaChado A Ferraris J Pérez PA Faraoni EY Pisera D. Role of GPER in the anterior pituitary gland focusing on lactotroph function. J Endocrinol. (2019) 240:99–110. doi: 10.1530/JOE-18-0402 30400046

[44] Rudolf FO Kadokawa H. Expression of estradiol receptor, GPR30, in bovine anterior pituitary and effects of GPR30 agonist on GnRH-induced LH secretion. Anim Reprod Sci. (2013) 139:9–17. doi: 10.1016/j.anireprosci.2013.04.003 23642498

[45] Veldhuis JD Takahashi PY Keenan DM Liu PY Mielke KL Weist SM. Age disrupts androgen receptor-modulated negative feedback in the gonadal axis in healthy men. Am J Physiol Endocrinol Metab. (2010) 299:E675–682. doi: 10.1152/ajpendo.00300.2010 PMC2957871 20682842

[46] Vaucher L Funaro MG Mehta A Mielnik A Bolyakov A Prossnitz ER. Activation of GPER-1 estradiol receptor downregulates production of testosterone in isolated rat Leydig cells and adult human testis. PLoS One. (2014) 9:e92425. doi: 10.1371/journal.pone.0092425 24736568 PMC3987996

[47] Lucas TF Pimenta MT Pisolato R Lazari MFM Porto CS. 17β-estradiol signaling and regulation of Sertoli cell function. Spermatogenesis. (2011) 1:318–24. doi: 10.4161/spmg.1.4.18903 PMC3271643 22332115

[48] Lucas TFG Royer C Siu ER Lazari MFM Porto CS. Expression and signaling of G protein-coupled estrogen receptor 1 (GPER) in rat sertoli cells. Biol Reprod. (2010) 83:307–17. doi: 10.1095/biolreprod.110.084160 20445128

[49] Ge L-C Chen Z-J Liu H-Y Zhang K-S Liu H Huang H-B. Involvement of activating ERK1/2 through G protein coupled receptor 30 and estrogen receptor α/β in low doses of bisphenol A promoting growth of Sertoli TM4 cells. Toxicol Lett. (2014) 226:81–9. doi: 10.1016/j.toxlet.2014.01.035 24495410

[50] Li Y-R Ren C-E Zhang Q Li J-C Chian R-C. Expression of G protein estrogen receptor (GPER) on membrane of mouse oocytes during maturation. J Assisted Reprod Genet. (2013) 30:227–32. doi: 10.1007/s10815-013-9942-z PMC3585672 23420106

[51] Prossnitz ER Maggiolini M. Mechanisms of estrogen signaling and gene expression *via* GPR30. Mol Cell Endocrinol. (2009) 308:32–8. doi: 10.1016/j.mce.2009.03.026 PMC2847286 19464786

[52] Casarini L Lazzaretti C Paradiso E Limoncella S Riccetti L Sperduti S. Membrane estrogen receptor (GPER) and follicle-Stimulating hormone receptor (FSHR) heteromeric complexes promote human ovarian follicle survival. iScience. (2020) 23:101812. doi: 10.1016/j.isci.2020.101812 33299978 PMC7702187

[53] Pavlik R Wypior G Hecht S Papadopoulos P Kupka M Thaler C. Induction of G protein-coupled estrogen receptor (GPER) and nuclear steroid hormone receptors by gonadotropins in human granulosa cells. Histochem Cell Biol. (2011) 136:289–99. doi: 10.1007/s00418-011-0846-7 21809103

[54] Hugues JN Cedrin-Durnerin I. Role of luteinizing hormone in follicular and corpus luteum physiology. Gynecologie Obstetrique Fertilite. (2000) 28:738–44. doi: 10.1016/s1297-9589(00)00005-9 11244636

[55] Pang Y Thomas P. Role of G protein-coupled estrogen receptor 1, GPER, in inhibition of oocyte maturation by endogenous estrogens in zebrafish. Dev Biol. (2010) 342:194–206. doi: 10.1016/j.ydbio.2010.03.027 20382141 PMC2874603

[56] Wen Y Zhan J Li C Li P Wang C Wu J. G-protein couple receptor (GPER1) plays an important role during ovarian folliculogenesis and early development of the Chinese Alligator. Anim Reprod Sci. (2023) 255:107295. doi: 10.1016/j.anireprosci.2023.107295 37422950

[57] Zhao H Ge J Wei J Liu J Liu C Ma C. Effect of FSH on E2/GPR30-mediated mouse oocyte maturation in vitro. Cell. Signalling. (2020) 66:109464. doi: 10.1016/j.cellsig.2019.109464 31704004

[58] Prossnitz ER Hathaway HJ. What have we learned about GPER function in physiology and disease from knockout mice? J Steroid Biochem Mol Biol. (2015) 153:114–26. doi: 10.1016/j.jsbmb.2015.06.014 PMC4568147 26189910

[59] Marjon NA Hu C Hathaway HJ Prossnitz ER. G protein-coupled estrogen receptor regulates mammary tumorigenesis and metastasis. Mol Cancer Research: MCR. (2014) 12:1644–54. doi: 10.1158/1541-7786.MCR-14-0128-T PMC4233188 25030371

[60] Jeng Y-J Kochukov M Watson CS. Combinations of physiologic estrogens with xenoestrogens alter calcium and kinase responses, prolactin release, and membrane estrogen receptor trafficking in rat pituitary cells. Environ Health: A Global Access Sci Source. (2010) 9:61. doi: 10.1186/1476-069X-9-61 PMC2967504 20950447

[61] Klenke U Constantin S Wray S. BPA directly decreases gnRH neuronal activity *via* noncanonical pathway. Endocrinology. (2016) 157:1980–90. doi: 10.1210/en.2015-1924 PMC4870872 26934298

[62] Adegoke EO Rahman MS Pang M-G. Bisphenols threaten male reproductive health *via* testicular cells. Front Endocrinol. (2020) 11:624. doi: 10.3389/fendo.2020.00624 PMC7518410 33042007

[63] Scaling AL Prossnitz ER Hathaway HJ. GPER mediates estrogen-induced signaling and proliferation in human breast epithelial cells and normal and Malignant breast. Hormones Cancer. (2014) 5:146–60. doi: 10.1007/s12672-014-0174-1 PMC4091989 24718936

[64] Jockers R Liu J. Editorial: endocrinology in cancer and aging. Front Endocrinol. (2021) 12:722929. doi: 10.3389/fendo.2021.722929 PMC8320168 34335482

[65] Berben L Floris G Wildiers H Hatse S. Cancer and aging: two tightly interconnected biological processes. Cancers. (2021) 13:1400. doi: 10.3390/cancers13061400 33808654 PMC8003441

[66] Santoro N Roeca C Peters BA Neal-Perry G. The menopause transition: signs, symptoms, and management options. J Clin Endocrinol Metab. (2021) 106:1–15. doi: 10.1210/clinem/dgaa764 33095879

[67] Santoro N Randolph JF. Reproductive hormones and the menopause transition. Obstetrics Gynecology Clinics North America. (2011) 38:455–66. doi: 10.1016/j.ogc.2011.05.004 PMC3197715 21961713

[68] Wright K Ly T Kriet M Czirok A Thomas SM. Cancer-associated fibroblasts: master tumor microenvironment modifiers. Cancers. (2023) 15:1899. doi: 10.3390/cancers15061899 36980785 PMC10047485

[69] Qureshi R Picon-Ruiz M Sho M Van Booven D Nunes de Paiva V Diaz-Ruano AB. Estrone, the major postmenopausal estrogen, binds ERa to induce SNAI2, epithelial-to-mesenchymal transition, and ER+ breast cancer metastasis. Cell Rep. (2022) 41:111672. doi: 10.1016/j.celrep.2022.111672 36384125 PMC9798480

[70] Rouhimoghadam M Lu AS Salem AK Filardo EJ. Therapeutic perspectives on the modulation of G-protein coupled estrogen receptor, GPER, function. Front Endocrinol. (2020) 11:591217. doi: 10.3389/fendo.2020.591217 PMC7719807 33329395

[71] Yuan J Liu M Yang L Tu G Zhu Q Chen M. Acquisition of epithelial-mesenchymal transition phenotype in the tamoxifen-resistant breast cancer cell: A new role for G protein-coupled estrogen receptor in mediating tamoxifen resistance through cancer-associated fibroblast-derived fibronectin and β1-integrin signaling pathway in tumor cells. Breast Cancer Research: BCR. (2015) 17:69. doi: 10.1186/s13058-015-0579-y 25990368 PMC4453053

[72] Wang Y Zhou BP. Epithelial-mesenchymal transition in breast cancer progression and metastasis. Chin J Cancer. (2011) 30:603–11. doi: 10.5732/cjc.011.10226 PMC3702729 21880181

[73] Xu E Xia X Jiang C Li Z Yang Z Zheng C. GPER1 silencing suppresses the proliferation, migration, and invasion of gastric cancer cells by inhibiting PI3K/AKT-mediated EMT. Front Cell Dev Biol. (2020) 8:591239. doi: 10.3389/fcell.2020.591239 33425895 PMC7793665

[74] Yang X-P Reckelhoff JF. Estrogen, hormonal replacement therapy and cardiovascular disease. Curr Opin Nephrol Hypertension. (2011) 20:133–8. doi: 10.1097/MNH.0b013e3283431921 PMC3123884 21178615

[75] Hasan M Browne E Guarinoni L Darveau T Hilton K Witt-Enderby PA. Novel melatonin, estrogen, and progesterone hormone therapy demonstrates anti-cancer actions in MCF-7 and MDA-MB-231 breast cancer cells. Breast Cancer: Basic Clin Res. (2020) 14:1178223420924634. doi: 10.1177/1178223420924634 PMC7318814 32636633

[76] Mørch LS Skovlund CW Hannaford PC Iversen L Fielding S Lidegaard Ø. Contemporary hormonal contraception and the risk of breast cancer. New Engl J Med. (2017) 377:2228–39. doi: 10.1056/NEJMoa1700732 29211679

[77] Chen B Ye P Chen Y Liu T Cha J-H Yan X. Involvement of the estrogen and progesterone axis in cancer stemness: elucidating molecular mechanisms and clinical significance. Front Oncol. (2020) 10:1657. doi: 10.3389/fonc.2020.01657 33014829 PMC7498570

[78] Ignatov A Ignatov T Weissenborn C Eggemann H Bischoff J Semczuk A. G-protein-coupled estrogen receptor GPR30 and tamoxifen resistance in breast cancer. Breast Cancer Res Treat. (2011) 128:457–66. doi: 10.1007/s10549-011-1584-1 21607586

[79] Ignatov T Claus M Nass N Haybaeck J Seifert B Kalinski T. G-protein-coupled estrogen receptor GPER-1 expression in hormone receptor-positive breast cancer is associated with poor benefit of tamoxifen. Breast Cancer Res Treat. (2019) 174:121–7. doi: 10.1007/s10549-018-5064-8 30478785

[80] Feitelson MA Arzumanyan A Kulathinal RJ Blain SW Holcombe RF Mahajna J. Sustained proliferation in cancer: Mechanisms and novel therapeutic targets. Semin Cancer Biol. (2015) 35 Suppl:S25–54. doi: 10.1016/j.semcancer.2015.02.006 PMC4898971 25892662

[81] Jala VR Radde BN Haribabu B Klinge CM. Enhanced expression of G-protein coupled estrogen receptor (GPER/GPR30) in lung cancer. BMC Cancer. (2012) 12:624. doi: 10.1186/1471-2407-12-624 23273253 PMC3557142

[82] Jia B Gao Y Li M Shi J Peng Y Du X. GPR30 promotes prostate stromal cell activation *via* suppression of ERα Expression and its downstream signaling pathway. Endocrinology. (2016) 157:3023–35. doi: 10.1210/en.2016-1035 27163843

[83] Xu T Ma D Chen S Tang R Yang J Meng C. High GPER expression in triple-negative breast cancer is linked to pro-metastatic pathways and predicts poor patient outcomes. NPJ Breast Cancer. (2022) 8:100. doi: 10.1038/s41523-022-00472-4 36042244 PMC9427744

[84] Gutiérrez-Almeida CE Santerre A León-Moreno LC Aguilar-García IG Castañeda-Arellano R Dueñas-Jiménez SH. Proliferation and apoptosis regulation by G protein-coupled estrogen receptor in glioblastoma C6 cells. Oncol Lett. (2022) 24:217. doi: 10.3892/ol.2022.13338 35720489 PMC9178726

[85] Tirado-Garibay AC Falcón-Ruiz EA Ochoa-Zarzosa A López-Meza JE. GPER: an estrogen receptor key in metastasis and tumoral microenvironments. Int J Mol Sci. (2023) 24:14993. doi: 10.3390/ijms241914993 37834441 PMC10573234

[86] Chan QKY Lam H-M Ng C-F Lee AYY Chan ESY Ng H-K. Activation of GPR30 inhibits the growth of prostate cancer cells through sustained activation of Erk1/2, c-jun/c-fos-dependent upregulation of p21, and induction of G(2) cell-cycle arrest. Cell Death Differentiation. (2010) 17:1511–23. doi: 10.1038/cdd.2010.20 PMC2897932 20203690

[87] Han N Heublein S Jeschke U Kuhn C Hester A Czogalla B. The G-protein-coupled estrogen receptor (GPER) regulates trimethylation of histone H3 at lysine 4 and represses migration and proliferation of ovarian cancer cells *in vitro* . Cells. (2021) 10:619. doi: 10.3390/cells10030619 33799631 PMC8001910

[88] Mattei AL Bailly N Meissner A. DNA methylation: A historical perspective. Trends Genetics: TIG. (2022) 38:676–707. doi: 10.1016/j.tig.2022.03.010 35504755

[89] Manjegowda MC Gupta PS Limaye AM. Hyper-methylation of the upstream CpG island shore is a likely mechanism of GPER1 silencing in breast cancer cells. Gene. (2017) 614:65–73. doi: 10.1016/j.gene.2017.03.006 28286086

[90] Weissenborn C Ignatov T Nass N Kalinski T Dan Costa S Zenclussen AC. GPER promoter methylation controls GPER expression in breast cancer patients. Cancer Invest. (2017) 35:100–7. doi: 10.1080/07357907.2016.1271886 28118074

[91] Tian S Zhan N Li R Dong W. Downregulation of G protein-coupled estrogen receptor (GPER) is associated with reduced prognosis in patients with gastric cancer. Med Sci Monitor: Int Med J Exp Clin Res. (2019) 25:3115–26. doi: 10.12659/MSM.913634 PMC6503750 31028714

[92] Liu Q Chen Z Jiang G Zhou Y Yang X Huang H. Epigenetic down regulation of G protein-coupled estrogen receptor (GPER) functions as a tumor suppressor in colorectal cancer. Mol Cancer. (2017) 16:87. doi: 10.1186/s12943-017-0654-3 28476123 PMC5418684

[93] Rong J Xie X Niu Y Su Z. Correlation between the RNA expression and the DNA methylation of estrogen receptor genes in normal and Malignant human tissues. Curr Issues Mol Biol. (2024) 46:3610–25. doi: 10.3390/cimb46040226 PMC11049367 38666956

[94] Calaf GM Ponce-Cusi R Aguayo F Muñoz JP Bleak TC. Endocrine disruptors from the environment affecting breast cancer. Oncol Lett. (2020) 20:19–32. doi: 10.3892/ol.2020.11566 PMC7286136 32565930

[95] Della Rocca Y Traini EM Diomede F Fonticoli L Trubiani O Paganelli A. Current evidence on bisphenol A exposure and the molecular mechanism involved in related pathological conditions. Pharmaceutics. (2023) 15:908. doi: 10.3390/pharmaceutics15030908 36986769 PMC10053246

[96] Dueñas-Moreno J Mora A Kumar M Meng X-Z Mahlknecht J. Worldwide risk assessment of phthalates and bisphenol A in humans: The need for updating guidelines. Environ Int. (2023) 181:108294. doi: 10.1016/j.envint.2023.108294 37935082

[97] Mariana M Castelo-Branco M Soares AM Cairrao E. Phthalates’ exposure leads to an increasing concern on cardiovascular health. J Hazardous Materials. (2023) 457:131680. doi: 10.1016/j.jhazmat.2023.131680 37269565

[98] Keshavarz-Maleki R Kaviani A Omranipour R Gholami M Khoshayand MR Ostad SN. Bisphenol-A in biological samples of breast cancer mastectomy and mammoplasty patients and correlation with levels measured in urine and tissue. Sci Rep. (2021) 11:18411. doi: 10.1038/s41598-021-97864-6 34531470 PMC8446007

[99] Molina L Figueroa CD Ehrenfeld P Figueroa CD Ehrenfeld P. Bisphenols and their interaction with gper-1: the invisible enemy behind breast cancer and its societal impact. In: IntechOpen, IntechOpen Limited, London United Kingdom (2024) 167–169. doi: 10.5772/intechopen.112880

[100] Stillwater BJ Bull AC Romagnolo DF Neumayer LA Donovan MG Selmin OI. Bisphenols and risk of breast cancer: A narrative review of the impact of diet and bioactive food components. Front Nutr. (2020) 7:581388. doi: 10.3389/fnut.2020.581388 33330580 PMC7710764

[101] Chen F-P Chien M-H. Lower concentrations of phthalates induce proliferation in human breast cancer cells. Climacteric: J Int Menopause Soc. (2014) 17:377–84. doi: 10.3109/13697137.2013.865720 24228746

[102] Kim IY Han SY Moon A. Phthalates inhibit tamoxifen-induced apoptosis in MCF-7 human breast cancer cells. J Toxicol Environ Health Part A. (2004) 67:2025–35. doi: 10.1080/15287390490514750 15513900

[103] Vandenberg LN Maffini MV Sonnenschein C Rubin BS Soto AM. Bisphenol-A and the great divide: A review of controversies in the field of endocrine disruption. Endocrine Rev. (2009) 30:75–95. doi: 10.1210/er.2008-0021 19074586 PMC2647705

[104] Li Z Ren Y Li X Wang W. KDM2A interacts with estrogen receptor α to promote bisphenol A and S-induced breast cancer cell proliferation by repressing TET2 expression. Ecotoxicology Environ Saf. (2023) 262:115132. doi: 10.1016/j.ecoenv.2023.115132 37315367

[105] Sheng Z Wang C Ren F Liu Y Zhu B. Molecular mechanism of endocrine-disruptive effects induced by Bisphenol A: The role of transmembrane G-protein estrogen receptor 1 and integrin αvβ3. J Environ Sci (China). (2019) 75:1–13. doi: 10.1016/j.jes.2018.05.002 30473274

[106] Reininger N Oehlmann J. Regrettable substitution? Comparative study of the effect profile of bisphenol A and eleven analogues in an *in vitro* test battery. Environ Sci Europe. (2024) 36:76. doi: 10.1186/s12302-024-00900-1

[107] Rao C Cao X Li L Zhou J Sun D Li B. Bisphenol AF induces multiple behavioral and biochemical changes in zebrafish (Danio rerio) at different life stages. Aquat Toxicol (Amsterdam Netherlands). (2022) 253:106345. doi: 10.1016/j.aquatox.2022.106345 36351319

[108] Harnett KG Chin A Schuh SM. BPA and BPA alternatives BPS, BPAF, and TMBPF, induce cytotoxicity and apoptosis in rat and human stem cells. Ecotoxicology Environ Saf. (2021) 216:112210. doi: 10.1016/j.ecoenv.2021.112210 33866271

[109] Lei B Sun S Zhang X Feng C Xu J Wen Y. Bisphenol AF exerts estrogenic activity in MCF-7 cells through activation of Erk and PI3K/Akt signals *via* GPER signaling pathway. Chemosphere. (2019) 220:362–70. doi: 10.1016/j.chemosphere.2018.12.122 30590302

[110] Mo Z Liu M Yang F Luo H Li Z Tu G. GPR30 as an initiator of tamoxifen resistance in hormone-dependent breast cancer. Breast Cancer Research: BCR. (2013) 15:R114. doi: 10.1186/bcr3581 24289103 PMC3978564

[111] Yin H Zhu Q Liu M Tu G Li Q Yuan J. GPER promotes tamoxifen-resistance in ER+ breast cancer cells by reduced Bim proteins through MAPK/Erk-TRIM2 signaling axis. Int J Oncol. (2017) 51:1191–8. doi: 10.3892/ijo.2017.4117 28902352

[112] Gassman NR. Induction of oxidative stress by bisphenol A and its pleiotropic effects. Environ Mol Mutagenesis. (2017) 58:60–71. doi: 10.1002/em.22072 PMC5458620 28181297

[113] Banerjee O Singh S Paul T Maji BK Mukherjee S. Centella asiatica mitigates the detrimental effects of Bisphenol-A (BPA) on pancreatic islets. Sci Rep. (2024) 14:8043. doi: 10.1038/s41598-024-58545-2 38580733 PMC10997607

[114] Menale C Mita D Diano N Diano S. Adverse effects of bisphenol a exposure on glucose metabolism regulation. Open Biotechnol J. (2016) 10:122–30. doi: 10.2174/1874070701610010122

[115] Rochester JR. Bisphenol A and human health: A review of the literature. Reprod Toxicol (Elmsford N.Y.). (2013) 42:132–55. doi: 10.1016/j.reprotox.2013.08.008 23994667

[116] LaKind JS Naiman DQ. Temporal trends in bisphenol A exposure in the United States from 2003-2012 and factors associated with BPA exposure: Spot samples and urine dilution complicate data interpretation. Environ Res. (2015) 142:84–95. doi: 10.1016/j.envres.2015.06.013 26121292

[117] Vandenberg LN Chahoud I Heindel JJ Padmanabhan V Paumgartten FJR Schoenfelder G. Urinary, circulating, and tissue biomonitoring studies indicate widespread exposure to bisphenol A. Environ Health Perspect. (2010) 118:1055–70. doi: 10.1289/ehp.0901716 PMC2920080 20338858

[118] Huo X Chen D He Y Zhu W Zhou W Zhang J. Bisphenol-A and female infertility: A possible role of gene-environment interactions. Int J Environ Res Public Health. (2015) 12:11101–16. doi: 10.3390/ijerph120911101 PMC4586663 26371021

[119] Rubin BS. Bisphenol A: An endocrine disruptor with widespread exposure and multiple effects. J Steroid Biochem Mol Biol. (2011) 127:27–34. doi: 10.1016/j.jsbmb.2011.05.002 21605673

[120] Zhang D Zhao K Han T Zhang X Xu X Liu Z. Bisphenol A promote the cell proliferation and invasion ability of prostate cancer cells *via* regulating the androgen receptor. Ecotoxicology Environ Saf. (2024) 269:115818. doi: 10.1016/j.ecoenv.2023.115818 38091676

[121] Focaccetti C Nardozi D Benvenuto M Lucarini V Angiolini V Carrano R. Bisphenol-A in drinking water accelerates mammary cancerogenesis and favors an immunosuppressive tumor microenvironment in BALB-neuT mice. Int J Mol Sci. (2024) 25:6259. doi: 10.3390/ijms25116259 38892447 PMC11172679

[122] Kundakovic M Champagne FA. Epigenetic perspective on the developmental effects of bisphenol A. Brain Behavior Immun. (2011) 25:1084–93. doi: 10.1016/j.bbi.2011.02.005 PMC3703316 21333735

[123] Rosenfeld CS. Transcriptomics and other omics approaches to investigate effects of xenobiotics on the placenta. Front Cell Dev Biol. (2021) 9:723656. doi: 10.3389/fcell.2021.723656 34631709 PMC8497882

[124] Xu KJ Loganathan N Belsham DD. Bisphenol S induces Agrp expression through GPER1 activation and alters transcription factor expression in immortalized hypothalamic neurons: A mechanism distinct from BPA-induced upregulation. Mol Cell Endocrinol. (2022) 552:111630. doi: 10.1016/j.mce.2022.111630 35569583

[125] Jackson E Shoemaker R Larian N Cassis L. Adipose tissue as a site of toxin accumulation. Compr Physiol. (2017) 7:1085–135. doi: 10.1002/cphy.c160038 PMC6101675 28915320

[126] Wang L Asimakopoulos AG Kannan K. Accumulation of 19 environmental phenolic and xenobiotic heterocyclic aromatic compounds in human adipose tissue. Environ Int. (2015) 78:45–50. doi: 10.1016/j.envint.2015.02.015 25749637

[127] Chelcea I Örn S Hamers T Koekkoek J Legradi J Vogs C. Physiologically based toxicokinetic modeling of bisphenols in zebrafish (Danio rerio) accounting for variations in metabolic rates, brain distribution, and liver accumulation. Environ Sci Technol. (2022) 56:10216–28. doi: 10.1021/acs.est.2c01292 PMC9301920 35797464

[128] Dalamaga M Kounatidis D Tsilingiris D Vallianou N Karampela I Psallida S. The role of endocrine disruptors bisphenols and phthalates in obesity: current evidence, perspectives and controversies. Int J Mol Sci. (2024) 25:675. doi: 10.3390/ijms25010675 38203845 PMC10779569

[129] Lucas A Herrmann S Lucas M. The role of endocrine-disrupting phthalates and bisphenols in cardiometabolic disease: The evidence is mounting. Curr Opin Endocrinology Diabetes Obes. (2022) 29:87–94. doi: 10.1097/MED.0000000000000712 PMC8915988 35034036

[130] Acramel A Jacquot Y. Deciphering of a putative GPER recognition domain in ERα and ERα36. Front Endocrinol. (2022) 13:943343. doi: 10.3389/fendo.2022.943343 PMC9279910 35846328

[131] Vella V De Francesco EM Lappano R Muoio MG Manzella L Maggiolini M. Microenvironmental determinants of breast cancer metastasis: focus on the crucial interplay between estrogen and insulin/insulin-like growth factor signaling. Front Cell Dev Biol. (2020) 8:608412. doi: 10.3389/fcell.2020.608412 33364239 PMC7753049

[132] Tang Z Li Q Cheng Q Mei M Song Y Du Z. G protein-coupled estrogen receptor 1 (GPER1) mediates aldosterone-induced endothelial inflammation in a mineralocorticoid receptor-independent manner. Int J Endocrinol. (2021) 2021:5575927. doi: 10.1155/2021/5575927 34239558 PMC8235990

[133] Hsu L-H Chu N-M Lin Y-F Kao S-H. G-protein coupled estrogen receptor in breast cancer. Int J Mol Sci. (2019) 20:306. doi: 10.3390/ijms20020306 30646517 PMC6359026

[134] Rocca A Braga L Volpe MC Maiocchi S Generali D. The predictive and prognostic role of RAS-RAF-MEK-ERK pathway alterations in breast cancer: revision of the literature and comparison with the analysis of cancer genomic datasets. Cancers. (2022) 14:5306. doi: 10.3390/cancers14215306 36358725 PMC9653766

[135] Cirillo F Talia M Santolla MF Pellegrino M Scordamaglia D Spinelli A. GPER deletion triggers inhibitory effects in triple negative breast cancer (TNBC) cells through the JNK/c-Jun/p53/Noxa transduction pathway. Cell Death Discovery. (2023) 9:353. doi: 10.1038/s41420-023-01654-0 37749101 PMC10520078

